# Rapid Determination of Chlorophyll and Pheophytin in Green Tea Using Fourier Transform Infrared Spectroscopy

**DOI:** 10.3390/molecules23051010

**Published:** 2018-04-26

**Authors:** Xiaoli Li, Ruiqing Zhou, Kaiwen Xu, Jie Xu, Juanjuan Jin, Hui Fang, Yong He

**Affiliations:** 1 College of Biosystems Engineering and Food Science, Zhejiang University, 866 Yuhangtang Road, Hangzhou 310058, China; xiaolili@zju.edu.cn (X.L.); ruiqingzhou@zju.edu.cn (R.Z.); 3110100219@zju.edu.cn (K.X.); jjjin@zju.edu.cn (J.J.); yhe@zju.edu.cn (Y.H.); 2 College of Biological Chemical Science and Engineering, Jiaxing University, Jiaxing 314001, China

**Keywords:** FT–IR spectroscopy, chlorophyll, pheophytin, green tea, quantification determination

## Abstract

The chlorophyll, pheophytin, and their proportions are critical factors to evaluate the sensory quality of green tea. This research aims to establish an effective method to determine the quantification of chlorophyll and pheophytin in green tea, based on Fourier transform infrared (FT–IR) spectroscopy. First, five brands of tea were collected for spectral acquisition, and the chlorophyll and pheophytin were measured using the reference method. Then, a relation between these two pigments and FT–IR spectroscopy were developed based on chemometrics. Additionally, the characteristic IR wavenumbers of these pigments were extracted and proved to be effective for a quantitative determination. Successively, non-linear models were also built based on these characteristic wavenumbers, obtaining coefficients of determination of 0.87, 0.80, 0.85 and 0.89; and relative predictive deviations of 2.77, 2.62, 2.26 and 3.07 for the four pigments, respectively. These results demonstrate the feasibility of FT–IR spectroscopy for the determination of chlorophyll and pheophytin.

## 1. Introduction

Color is one of the most important quality indices of green tea, which frequently influences and determines the orientation of people’s consumption [1]. In the process of making green tea, a fresh tea leaf is fixed at a high temperature, which deactivates a variety of enzymes, and keeps the green color of the tea. Thus, the color of green tea is dominated by green, which is mainly caused by its composition of chlorophyll, including chlorophyll a (Chl-a) and chlorophyll b (Chl-b) [2]. The color of chlorophyll is determined by four nitrogen atoms, which form the central metal binding pocket of the tetrapyrrole-derived macrocycles, usually occupied by a Mg^2+^ ion [3]. In addition, the macrocyclic π-electron system, the central metal ion, and the peripheral substituents have a strong effect on the ground- and excited-state parameters, the solar energy conversion, and the pigment photostability [3]. Moreover, the species of the central metal atoms affect the stability and toxicity of chlorophyll [4]. Different chlorophyll components have an effect on photosystem II, which reflects distinct spectroscopic features of the regions of the keto and ester C=O stretches and the chlorin ring vibrations of Pheo [5]. It is worth noting that chlorophyll is very sensitive and unstable when exposed to light, heat, and oxygen [6], which lead to a decomposition of chlorophyll to form pheophytin during the tea-making process or storage. Two olive brown pigments of pheophytin a (Phe-a) and pheophytin b (Phe-b), as the main chlorophyll derivatives, are formed by losing Mg^2+^ from the porphyrin ring under acid conditions [7]. The structural properties of the porphyrin pigments considerably affect the absorption spectra in the UV–VIS region [8]. In detail, the chlorophylls exhibit peaks at 660 nm (Q band), and 430 and 453 nm (Soret bands), while the peaks of pheophytins switch to 666 nm (Q band) and 434 nm (Soret bands) [8]. Some food processes could also alter the spectral properties of food due to the changes of the total amount of chlorophyll and the proportion of various chlorophyll ingredients; e.g., a high hydrostatic pressure could cause hypsochromic shift of the peak position in the pre-ethylene stages, but in the post-ethylene stages, the shift would decrease due to the increase of the pheophytin [9]. The conversion of chlorophyll to pheophytin turns the color of green tea to olive brown [10], which largely affects the sensory quality and degree of deterioration of green tea. Moreover, the antioxidant and anti-inflammatory activities would decrease during the decomposition of chlorophyll to pheophytin [8]. Thus, the amount of chlorophyll and pheophytin, and their proportions, could be used as critical factors to evaluate the color and shelf life of green tea [11].

Over the last few decades, various approaches have been employed to measure tea quality. For example, traditional chemical analyses, such as spectrophotometry [12], high-performance liquid chromatography (HPLC) [13], gas chromatography-mass spectrometry [14], inductively coupled plasma mass spectrometry [15], and so on. These chemical analyses are precise, but they require complex sample preparations. To date, non-invasive approaches (e.g., infrared spectroscopy) have been proposed for the rapid detection of the internal attributes of tea products. Several studies have been successfully carried out to apply infrared spectroscopy in the qualitative and quantitative analysis of some active compositions in tea. Li et al. [16] and Paradkar and Irudayaraj [17] demonstrated the feasibility of using infrared spectroscopy techniques to predict the amount of polyphenols and caffeine in tea. Chen et al. [18] and Dey et al. [19] applied FT–NIR spectroscopy technique to discriminate the geographical origins of Chinese green tea. Panigrahi et al. [1] used diffuse reflectance spectroscopy to discriminate different black tea grades. Bunghez et al. [20] and Hashimoto et al. [21] applied FT–IR to quantify the amount of lycopene, ethanol, and sugar in plants.

To the best of our knowledge, a few spectroscopic studies have been conducted to determine, nondestructively, chlorophyll and pheophytin in green tea as regards sensor quality. Thus, this paper applied FT–IR spectroscopy to determine, quantitatively, chlorophyll and pheophytin in green tea. In addition, a FT–IR spectroscopy spectrometer has a high resolution and contains bands of large wavelengths. Additionally, it contains plenty of redundancy variables and multi-colinearity [22], which will lead to a time-consuming and inefficient modeling process. Therefore, characteristic IR wavenumbers were extracted to simplify the determination models.

The objectives of this study were: (1) to investigate the potential of FT–IR spectroscopy for the determination of chlorophyll and pheophytin in green tea; and (2) to explore FT–IR characteristic wavenumbers of chlorophyll and pheophytin, based on chemometric approaches.

## 2. Results and Discussion

### 2.1. Overview of FT–IR Spectra

**Figure 1:**
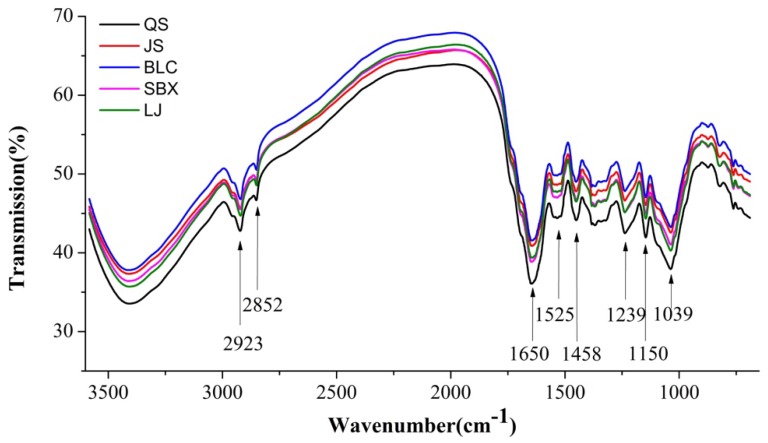
Averaged spectra of five green tea brands in the range of 3582–689 cm^−1^.

In this study, five famous brands of green tea, Queshe (QS), Jingshan (JS), Biluochun (BLC), Sanbeixiang (SBX), and Longjing (LJ), were used in the experiments. Figure 1 shows the spectral profiles of all samples, from 3582 to 689 cm^−1^, and the main absorption peaks are marked with their wavenumber values. It can be found that the spectral profiles of the samples, for different brands, are quite similar. The main large peaks of 1650, 1525, 1458, 1239, 1150, and 1039 cm^−1^ could be detected in the fingerprint region of the FT–IR spectra. In detail, the peak around 1650 cm^−1^ could be the vibration of the amide I (N–H bending), the peak around 1525 cm^−1^ is related to the band of the amide II, and the peak of 1239 cm^−1^ is assigned to the amide III band [23,24]. The peak at 1150 cm^−1^ is assigned to the anti-symmetric stretching of the C–O–C bridge [25]. The spectral responses near 2923 cm^−1^ and 2852 cm^−1^ are associated with anti-symmetric and symmetric C–H stretching vibrations of methylene (CH_2_) groups, respectively [26]. In addition, the presence of the band at 1458 cm^−1^ is caused by the symmetric bending mode of methylene groups [27].

### 2.2. Overview of Chlorophyll and Pheophytin of Tea Samples

**Figure 2:**
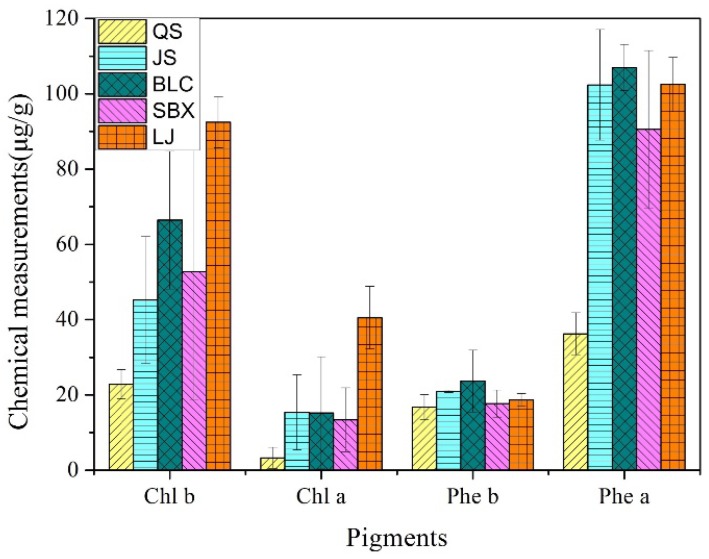
Mean amount of the lipid-soluble pigments in five tea brands.

The distribution of chlorophyll (Chl) and pheophytin (Phe) concentrations of all samples, for the five brands, are shown in Figure 2. The concentration of Phe-a is obviously higher than that of Chl-a for all five brands, indicating that most of the Chl-a was converted to Phe-a. While the concentration of Phe-b is obviously lower than that of Chl-b for all five brands, indicating that Chl-b was relatively stable compared to Chl-a. The color of green tea is a comprehensive reflection of a variety of pigmented substances. The QS brand has the lowest concentrations of all pigments compared to the other brands. The LJ brand has a relatively higher concentration of Chl-b and Chl-a, so the color of LJ is more yellowish-green.

### 2.3. Quantitative Determination of Chlorophylls and Pheophytin

As the pigments of chlorophylls and pheophytins play an important role in evaluating the quality of green tea, a fast quantitative determination of the pigments would be very helpful for quality control in the postharvest production of green tea.

#### 2.3.1. Sample Division

Before the quantitative analysis of the chlorophylls (Chl-a and Chl-b) and pheophytin (Phe-a and Phe-b), a total of 135 tea samples were partitioned into a validation set and a prediction set, based on the Kennard-Stoned (K-S) algorithm. The objective of K-S is to select a representative subset as a training set, so the numerical range of the training samples can be evenly spread throughout the sample space. In this study, 90 samples were selected to train and the others 45 samples were taken to evaluate the predictive ability of the training model. The full-cross validation method was used to validate the stability of the model. Table 1 shows the statistical results of the Chl-b, Chl-a, Phe-b and Phe-a amounts for all three sample sets. It can be found that the training set could cover the concentration ranges of all four pigments.

Chlorophyll is a dominant pigment of fresh green tea leaves, while the pheophytin amount is very low. During the storage process, acidity increases, which causes the loss of the magnesium ion in the chlorophylls and produces an olive-brown solid (pheophytin) [4]. As shown in Table 1, there is a distinct variation in the amount of the four pigments. The amount of Chl-b is about double that of Chl-a. In the meantime, comparing the amounts of Phe-a and Phe-b in green tea products, it could be found that Phe-a is about three times higher than Phe-b. Lee et al. [2] found that the proportion of Chl-b and Chl-a increased in tea samples after the drying process, and there was a negatively proportional relationship between either Chl-a and Phe-a or Chl-b and Phe-b. This illustrates that Chl-a was more easily degraded and converted into Phe-a during the tea harvesting and processing compared to Chl-b.

#### 2.3.2. Spectra Pretreatment

The Partial Least Squares (PLS) algorithm was adopted to establish the lipid-soluble pigment models. In the spectra acquisition process, it is quite common that the collected spectra may contain biased and noisy signals affected by light scattering, baseline drift, and the length variation of the light path. Therefore, to minimize the interference in the original spectra, standard normal variate (SNV) transformation was applied, as a pretreatment method, to the original spectra. Full spectra PLS models were built based on the pretreated spectra, as well as the original ones. Table 2 shows the results of these models.

As shown in Table 2, SNV provides a preferable result relative to that based on the original data. In detail, the R^2^_P_ of Chl-b, Chl-a, and Phe-b were improved to 0.76, 0.78 and 0.91, respectively. The models based on SNV are more stable when the gap between the R^2^_P_ and R^2^_V_ decreases. Consequently, the SNV pretreatment was used to build models 1-A, 1-B, 1-C and 1-D for Chl-b, Chl-a, Phe-b, and Phe-a, respectively.

**Figure 3:**
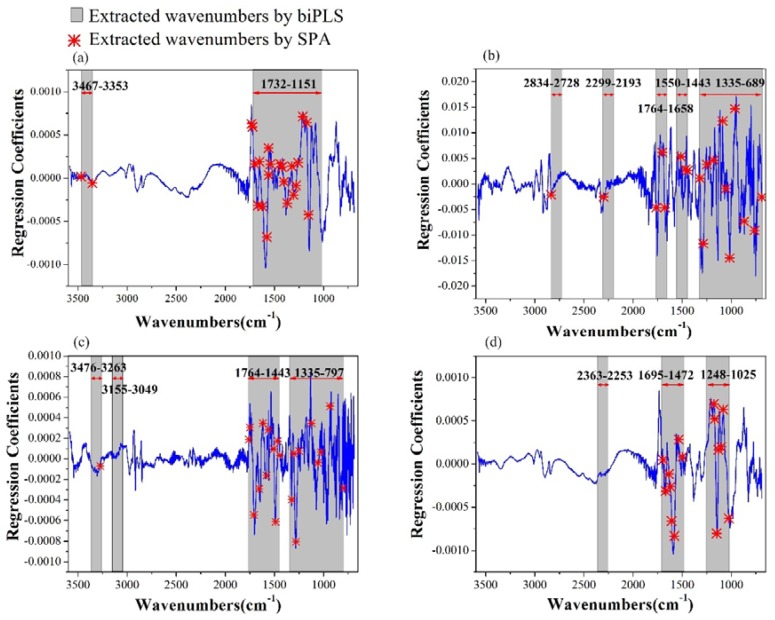
Weighted regression coefficients of PLS models and the extracted wavenumbers by biPLS and SPA for (**a**) Chl-b; (**b**) Chl-a; (**c**) Phe-b; (**d**) Phe-a.

The regression coefficients of the PLS models were obtained, as shown in Figure 3. The wavenumbers with large absolute values of weighted regression coefficients are mainly located in the IR fingerprint area (1300–700 cm^−1^), which indicates that the fingerprint area was more important and relevant for the researched pigments [28].

#### 2.3.3. Selection of Characteristic Wavenumbers

FT–IR spectroscopy provides information about the internal ingredients of tea, with a wide range of wavenumbers. In this research, the former models, listed in Table 2, were all built based on the full wavenumber range of 3583–689 cm^−1^. However, the spectra data of the full range may contain irrelevant information, which frequently worsens the stability and accuracy of a model. Concerning the development of an online and rapid determination of the amount of Chl-b, Chl-a, Phe-b and Phe-a in tea, these models should be further simplified. In addition, to explore the mechanism between these pigments and their FT–IR spectra, a wavenumber selection method of biPLS combined with SPA was used to select the characteristic wavenumbers.

Taking Chl-b as an example, through biPLS, the full spectra from 3583–689 cm^−1^ were equally split into 27 sub-intervals, and each interval had about 143 wavenumbers. Then, the PLS model was developed for each combination in these intervals. The model based on six intervals contained 720 variables and had the lowest RMSE value. Thus, these 720 wavenumbers were chosen to build the best PLS model for the determination of Chl-b. The distribution of the extracted wavenumbers is shown in Figure 3. These wavenumbers, which had a large absolute value of the regression coefficients, were chosen. The biPLS models results for the four pigments (models 2-A, 2-B, 2-C, 2-D) are shown in Table 3. The biPLS models were enhanced by varying degrees, compared to the original PLS models. In the case of Chl-b, the R^2^_p_ was improved from 0.76 to 0.80 compared to the PLS models in Table 2. The biPLS models progressed on the values of R^2^ and RPD, while the gap between validation and prediction was smaller. Thus, the biPLS models obtained a higher precision and were more stable and simple.

**Figure 4:**
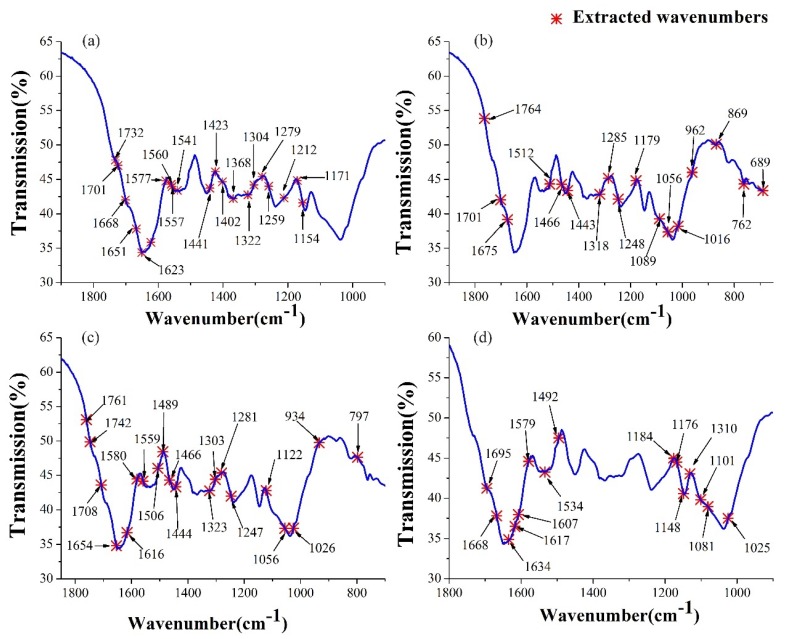
Extracted wavenumbers by biPLS and SPA for (**a**) Chl-b; (**b**) Chl-a; (**c**) Phe-b; (**d**) Phe-a.

Based on biPLS, the selected wavenumbers used for the regression modeling were reduced to about a half in dimension. However, the successive wavenumbers selected by biPLS were not optimal because they still contained some redundant and collinear wavenumbers. Thus, the SPA method was proposed in this work to select the characteristic wavenumbers and solve the collinear problems of the model based on biPLS. For the four pigments, the distributions of the characteristic wavenumbers selected by SPA are shown in Figure 4. It indicated that most of the selected wavenumbers were located at the peaks and valleys of the coefficient curves, which played an important role in the determination of the models. The selected wavenumbers—mainly located in the peaks and valleys of the coefficient curves—indicate a dominant role in the determination of the models. 

In detail, the wavenumbers around 1730 and 1700 cm^−1^ are related to the ester C=O and keto C=O band of the neutral pheophytin and chlorophyll, respectively; while the bands around 1616 cm^–1^ are related to the C=C in chlorophyll and its derivatives [29,30]. The bands from 1580 to 1510 cm^−1^ are contributed to the N–H stretching of the amide II [31]. The bands from 1490 to 1440 cm^−1^ are the features of the chlorine and quinone ring bands [5], including the C=O at 1489 cm^−1^, C–N stretching at 1466 cm^−1^ and C–H stretching at 1444 cm^−1^. The region from 1320 and 1300 cm^−1^ are contributed to the amide III vibration and the CH_2_ wagging modes [32]. The wavenumbers around 1280 cm^−1^ are assigned to the stretching vibrations of C–O for the ester groups [8]. The bands at 1154 and 1148 cm^−1^ contributed to the coupled C–C and C–O vibrations, to indicate carbohydrates [33]. From the perspective of the characteristic bands, the quantitative models are based on both, the bands associated with chlorophyll and its derivatives; and other carbohydrates and amides, which proves that comprehensive information would help to establish a better prediction model.

Comparing to Model 2-A–2-D in Table 3, both the validation value and the RPD of Model 3-A–3-D were relatively lower as shown in Table 4. Except for the RPD value of Chl-b, which increases to 2.47 (from 2.22 of Model 2-A). These results indicate that the performance of Model 3 based on the combination of biPLS and SPA was slightly worse than that of Model 2, based on biPLS. When Model 3 is compared to Model 1, the R^2^_P_ for the Chl-b prediction was increased from 0.76 to 0.83. In addition, the most remarkable achievement of the SPA method was the reduction of the spectral variables from about 1000 to 20. The models based on the combination of biPLS and SPA obtained comparable results with the full range models. It can be concluded that this wavenumber selection method achieved to eliminate the useless and irrelevant wavenumbers, as well as to maintain a high accuracy of the models. It demonstrates that the extracted wavenumbers are successful in generating a higher precision and more stable models with good interpretability of the FT–IR spectroscopy.

#### 2.3.4. Establishment of Nonlinear Determination Models

**Figure 5:**
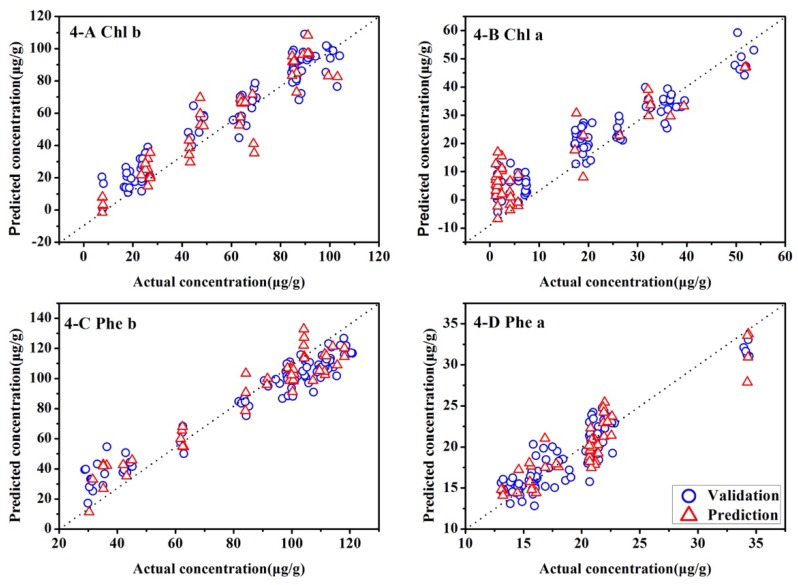
Scatter plots of measured vs. predicted concentrations of the LS-SVM, (**A**) Model 4-A of Chl-b; (**B**) Model 4-B of Chl-a; (**C**) Model 4-C of Phe-b; (**D**) Model 4-D of Phe-a.

To further improve the accuracy and operation time of the models for the four pigments in tea, LS-SVM was proposed to build models of nonlinear determination based on the characteristic wavenumbers. In this study, RBF kernel was used as the kernel function of LS-SVM. Before establishing the nonlinear model, a two-dimensional minimization grid search method was applied to determine two optimal parameters; namely, the regularization parameter gamma (γ) and the RBF kernel functional parameter sigma squared (σ^2^). The ranges of γ and σ^2^ were respectively set as 1–10^6^ and 1–10^4^.After the process of optimization, the corresponding results of the LS-SVM model for each pigment were obtained, as shown in Table 5 and Figure 5. As shown in Table 5, the nonlinear Models 4-A–4-D are greatly optimized, achieving the great performance for all sample sets. The RPD values of Model 4 are all above 2. Models 4-A–4-D satisfy the demand of reducing the modeling time and improving the accuracy of the models at the same time, so they are the ideal models to determine the amount of Chl-b, Chl-a, Phe-b and Phe-a in green tea.

## 3. Materials and Methods 

### 3.1. Sample Preparation

In this study, five brands of tea were bought from a local market (Hangzhou Zhongming Tea Co., Ltd., Hangzhou, China), which included Queshe tea (QS, from Huangshan, Anhui, China), Jingshan tea (JS, from Hangzhou, Zhejiang, China), Biluochun tea (BLC, from Suzhou, Jiangsu, China), Sanbeixiang tea (SBX, from Wenzhou, Zhejiang, China) and Longjing tea (LJ, from Hangzhou, Zhejiang, China). For each brand, three grades of H, M and L levels were collected. In detail, H was the best grade with the highest price, M was the mid-range, and L was the low-grade with the lowest price. Nine samples were gathered for each grade and brand, so a total of 135 tea samples were obtained.

First, each tea sample, with about 5 g, were successively milled for 30 s using a grinder (FW100, Ty, instrument Co., Ltd., Shanghai, China). After that, the ground samples were sieved through a 60-mesh sifter. Then, 0.1 g of the sieved sample was mixed adequately with 4.9 g of KBr. Finally, 0.1 g of the mixed samples were converted into tablets for FT–IR spectroscopy scanning. Meanwhile, another 0.5 g of the sieved sample was prepared for the HPLC measurement of chlorophyll and pheophytin. The temperature was kept at about 25 °C throughout the experiment.

### 3.2. FT–IR Spectroscopy Acquisition

In this study, a Fourier transform infrared (FT–IR) spectrometer (Thermo Scientific^TM^ Nicolet^TM^ iS^TM^ 10, Madison, WI, USA) was adopted for collection of samples’ FT–IR spectroscopy in transmittance mode. The range of this spectrometer was 7800–349 cm^−1^, with a resolution of 4 cm^−1^ and the sampling interval of 0.96 cm^−1^. Each sample was scanned 32 times and an average spectrum was taken as a representative of the sample. The whole operation was processed at about 25 °C and in a dark environment.

### 3.3. HPLC Measurement Conditions

As a reference method for the measurement of chlorophyll and pheophytin, the high-performance liquid chromatography (HPLC) conditions are shown as follow. First, a tea powder (0.5 g) sample was mixed with 5 mL of pigment extraction solution (acetone: water = 4:1; *v*/*v*) in a 10 mL centrifuge tube. Then, the tube was placed at 4 °C for 2 h and centrifuged for 5 min at the rotate speed of 5000 revolutions per minute. The supernatant was collected and 5 mL of pigment extraction solution was added to the pellet to re-extract the pigments according to the above procedure. The supernatants of the two steps were combined and filtered with a 0.45 μm organic filter membrane for HPLC analysis.

A Shimadzu LC-20AD HPLC system (Shimadzu, Kyoto, Japan) coupled with a UV–Visible detector (wavelength range: 190–600 nm) was used for the detection of the tea acetone extraction. The HPLC conditions were: Diamonsil C18 column (particle size: 5 μm, 250 mm × 4.6 mm) (Beijing di Technology Co., Ltd., Beijing, China), mobile phase A was: acetonitrile: acetic acid: distilled water (6:1:193; *v*/*v*/*v*), mobile phase B was: acetonitrile: methyl alcohol (1:2; *v*/*v*). The linear gradient elution procedure was: 0–10 min, 80% mobile phase B; 10–20 min, 80% mobile phase B increased to 100% mobile phase B; 20–100 min, 100% mobile phase B; 100–110 min, 100% mobile phase B decreased to 80% mobile phase B; 110–120 min, 80% mobile phase B. Flow rate was 1 mL min^−1^. Injection volume was 50 μL. The UV detection wavenumber was 450 nm and the column temperature was set at 35 °C.

### 3.4. Chemometric Methods

#### 3.4.1. Establishment of Quantitative Determination Models

A partial least square (PLS) regression was applied in this research to establish a quantitative determination model of chlorophyll and pheophytin of green tea. PLS is a widely-used bilinear modeling method to find the fundamental relations between the spectral data and known chemical components [16,34].

Least squares support vector machine (LS-SVM) was used to establish a nonlinear determination model. LS-SVM is a novel statistical learning algorithm which can interpret the linear or nonlinear relationships between the original independent information and its properties [35,36].

The determination model was evaluated with several indexes, including root mean square error (RMSE), the coefficient of determination (R^2^) and the relative predictive deviation (RPD). Generally, a good model should have a low RMSE value and a high value of R^2^ and RPD. RPD is calculated to assess the predictive ability of the determination model. RPD values less than 1.0 indicates very poor model or predictions and it is not recommended; between 1.0 and 1.4 indicates poor model or predictions, where only high and low values can be distinguished; between 1.4 and 1.8 indicates fair model or predictions, which may be used to evaluate and correlations; between 1.8 and 2.0 indicate good model or predictions, where the quantitative predictions are possible; between 2.0 and 2.5 indicates very good quantitative model or predictions; greater than 2.5 indicates excellent performance of the model or predictions [37].

#### 3.4.2. Extraction of Characteristic Wavenumbers

In this research, a backward interval partial least square (biPLS) was integrated into a successive project algorithm (SPA), to select characteristic wavenumbers for chlorophyll and pheophytin. Thus, these characteristic wavenumbers would reveal the mechanism of FT–IR spectral detection of these pigments. The biPLS was made by Takayama et al. [38] based on iPLS algorithm, and iPLS is a graphically oriented approach for local progression modeling of spectral data based on PLS [39]. The biPLS can extract spectral features with high efficiency.

The SPA is carried out following biPLS, to extract a smaller representative set of spectral variables. SPA is a forward variable selection method that employs simple projection operations into a vector space to find subsets of variables with minimal collinearity [12].

The PLS was carried out using Unscrambler 9.7^®^ (CAMO S/A) software. The LDA, iPLS, SPA and LS-SVM algorithms were performed with Matlab^®^ 2014b (Mathworks Inc., Natick, MA, USA) software.

## 4. Conclusions

These results indicate that it was feasible to determine Chl-b, Chl-a, Phe-b and Phe-a in green tea based on FT–IR spectroscopy. Therefore, the study provided a superior alternative to rapidly provide a sensory evaluation index of green tea.

By means of the combination of the method of wavenumbers selection and the LS-SVM algorithm, a quantification relationship was established between the FT–IR spectra and the four pigments. Through wavenumber selection of the biPLS and SPA, about twenty wavenumbers were selected to establish the determination models which reduced the computation complexity. The generalization of the models performed remarkably well with a high predicted accuracy (PRD of 2.77, 2.62, 2.26 and 3.07 for Chl-b, Chl-a, Phe-b and Phe-a, respectively). In addition, the overall results have sufficiently demonstrated that the proposed FT–IR spectroscopy technique, coupled with chemometric methods, was reliable and efficient for chlorophyll and pheophytin measurement. The extracted wavenumbers allowed us a good interpretation of the spectroscopy and could be used to develop a simple, low-cost, and efficacious instrument.

Overall, FT–IR spectroscopy coupled with chemometric methods can rapidly determine chlorophyll and pheophytin, which could provide a new auxiliary method for quality control and process monitoring in the green tea industry.

## Figures and Tables

**Table 1:** Statistical analyses of the amounts of the four pigments in the validation and prediction sets.

Sample Set	Pigment	Mean (μg/g)	SD (μg/g)	Range (μg/g)
Training	Chl-b	57.98	30.32	7.50–104.06
	Chl-a	20.58	15.23	1.19–53.61
	Phe-b	19.28	4.72	13.19–34.41
	Phe-a	88.92	29.53	28.84–120.96
Prediction	Chl-b	51.93	29.57	7.51–103.11
	Chl-a	21.79	15.18	1.20–52.01
	Phe-b	20.39	5.38	13.19–34.31
	Phe-a	85.12	28.52	30.33–118.11

SD: standard deviation.

**Table 2:** The PLS model results based on pretreatment methods.

Pigment	Model	Pretreatment	Validation		Prediction	
			RMSEV (μg/g)	R^2^_V_	RMSEP (μg/g)	R^2^_P_
Chl-b	1-A	Ori	9.69	0.90	14.86	0.74
		SNV	9.55	0.90	14.38	0.76
Chl-a	1-B	Ori	4.16	0.92	7.92	0.74
		SNV	4.46	0.91	7.05	0.78
Phe-b	1-C	Ori	1.95	0.83	1.87	0.88
		SNV	1.85	0.86	1.62	0.91
Phe-a	1-D	Ori	11.40	0.83	9.49	0.89
		SNV	8.73	0.92	9.23	0.89

RMSEV: RMSE of validation set; R^2^_V_: R^2^ of validation set; RMSEP: RMSE of prediction set; R^2^_P_: R^2^ of prediction set.

**Table 3:** The biPLS model results based on the extracted wavenumbers.

Set	Model	2-A	2-B	2-C	2-D
	Pigment	Chl-b	Chl-a	Phe-b	Phe-a
	Wavenumbers	720	1115	1225	579
Validation	RMSEC (μg/g)	8.43	4.44	1.60	7.32
	R^2^_V_	0.92	0.92	0.89	0.94
Prediction	RMSEP (μg/g)	13.30	6.98	1.64	12.17
	R^2^_P_	0.80	0.78	0.91	0.81
	RPD	2.22	2.16	3.23	2.34

**Table 4:** The model results based on wavenumbers selected by the combination of biPLS and SPA.

Set	Model	3-A	3-B	3-C	3-D
	Pigment	Chl-b	Chl-a	Phe-b	Phe-a
	Wavenumbers	19	19	21	14
Validation	RMSEC (μg/g)	8.70	5.28	1.89	8.15
	R^2^_V_	0.92	0.88	0.85	0.92
Prediction	RMSEP (μg/g)	11.94	8.38	2.12	9.76
	R^2^_P_	0.83	0.68	0.84	0.88
	RPD	2.47	1.80	2.50	2.92

**Table 5:** The LS-SVM model results based on the characteristic wavenumbers.

Set	Model	4-A	4-B	4-C	4-D
	Pigment	Chl-b	Chl-a	Phe-b	Phe-a
	Wavenumbers	19	19	21	14
Validation	RMSEC (μg/g)	8.30	4.61	1.89	6.64
	R^2^_V_	0.93	0.91	0.84	0.95
Prediction	RMSEP (μg/g)	10.66	6.69	2.02	9.28
	R^2^_P_	0.87	0.80	0.85	0.89
	RPD	2.77	2.26	2.62	3.07
	Slope	1.00	0.83	0.82	1.07
	Bias	1.46	3.44	3.49	-3.41
